# Unique Haploinsufficient Role of the MicroRNA-Processing Molecule *Dicer1* in a Murine Colitis-Associated Tumorigenesis Model

**DOI:** 10.1371/journal.pone.0071969

**Published:** 2013-09-02

**Authors:** Takeshi Yoshikawa, Motoyuki Otsuka, Takahiro Kishikawa, Akemi Takata, Motoko Ohno, Chikako Shibata, Young Jun Kang, Haruhiko Yoshida, Kazuhiko Koike

**Affiliations:** 1 Department of Gastroenterology, Graduate School of Medicine, The University of Tokyo, Tokyo, Japan; 2 Japan Science and Technology Agency, PRESTO, Kawaguchi, Saitama, Japan; 3 Department of Immunology and Microbial Science, The Scripps Research Institute, La Jolla, California, United States of America; National Institute for Viral Disease Control and Prevention, CDC, China, China

## Abstract

A widespread downregulated expression of microRNAs (miRNAs) is commonly observed in human cancers. Similarly, deregulated expression of miRNA-processing pathway components, which results in the reduction of global miRNA expression, may also be associated with tumorigenesis. Here, we show that specific ablation of *Dicer1* in intestinal epithelial cells accelerates intestinal inflammation-associated tumorigenesis. This effect was apparent only when a single copy of *Dicer1* was deleted, but not with complete *Dicer1* ablation. DICER expression and subsequent mature miRNA levels were inversely correlated with the number of intact *Dicer1* alleles. Because the expression levels of DICER were retained in tumors and its surrounding tissues even after induction of colitis-associated tumors, the effects of *Dicer1* deletion were cell-autonomous. Although the expression levels of representative oncogenes and tumor suppressor genes were in most cases inversely correlated with the expression levels of DICER, some genes were not affected by *Dicer1* deletion. Thus, deregulating the delicate balance between the expression levels of tumor-promoting and -suppressive genes may be crucial for tumorigenesis in this unique haploinsufficient case.

## Introduction

While microRNAs (miRNAs) can function as both tumor suppressors and oncogenes in tumor development [1]–[3], widespread downregulated expression of miRNAs promotes cellular transformation and tumorigenesis and is commonly observed in human cancers [1], [4], [5]. Similar to miRNAs, deregulated expression of miRNA processing pathway components can potentially modify miRNA expression profiles and may be associated with subsequent tumorigenesis and tumor prognosis [6]–[11].

Dicer is a ribonuclease (RNase) III enzyme required for pre-miRNA processing that originates from endogenous single-stranded hairpin- or repeat-associated precursors [12]. A defect in *Dicer* is one possible mechanism of global downregulated expression of mature miRNAs. Mutations in *Dicer* were identified in pediatric tumor pleuropulmonary blastoma, Sertoli-Leydig cell tumors, or other tumors [13], [14] and downregulation of DICER is associated with poor prognosis of ovarian cancers and other tumors [11], [15], [16]. These clinical observations, together with experimental results showing the global loss of mature miRNAs induced by Dicer knockdown *in vitro* and *in vivo*, demonstrates that Dicer is functionally relevant to oncogenesis [17].

Subsequent studies using mouse models with tissue-specific *Dicer1* ablation reported that a single allele of *Dicer1* in mice leads to oncogenesis in a lung cancer model caused by a K-ras protooncogene mutation [18], a retinoblastoma model caused by mutations in the tumor suppressor *RB* gene [19], and a lymphoma model caused by Eµ-myc [20]. In these models, monoallelic, but not complete, loss of *Dicer* alleles enhanced tumor formation. In addition, although frequent deletion of a *Dicer* allele has been reported, inactivation of the second wild-type allele has not [18].

Therefore, it has been proposed that Dicer may be unique among classical haploinsufficient tumor suppressor genes since only partial, but not complete, loss promotes tumor development [21]. However, it remains possible that this unique haploinsufficient function of *Dicer* may be tissue-specific and related to tissue-specific expression of miRNAs and miRNA processing pathway-related genes [20]. It is also unknown whether previous results were dependent on tumorigenesis models caused by enforced mutations in oncogenes or tumor suppressor genes.

To clarify the role of *Dicer*, we examined mice with intestinal epithelial cell-specific *Dicer1* ablation in colitis-associated tumorigenesis. We were particularly interested in the possibility that different effects may result from monoallelic and biallelic ablation of *Dicer1*.

## Results

### Intestinal epithelial cell-specific *Dicer1*-mutant mice

**Figure 1:**
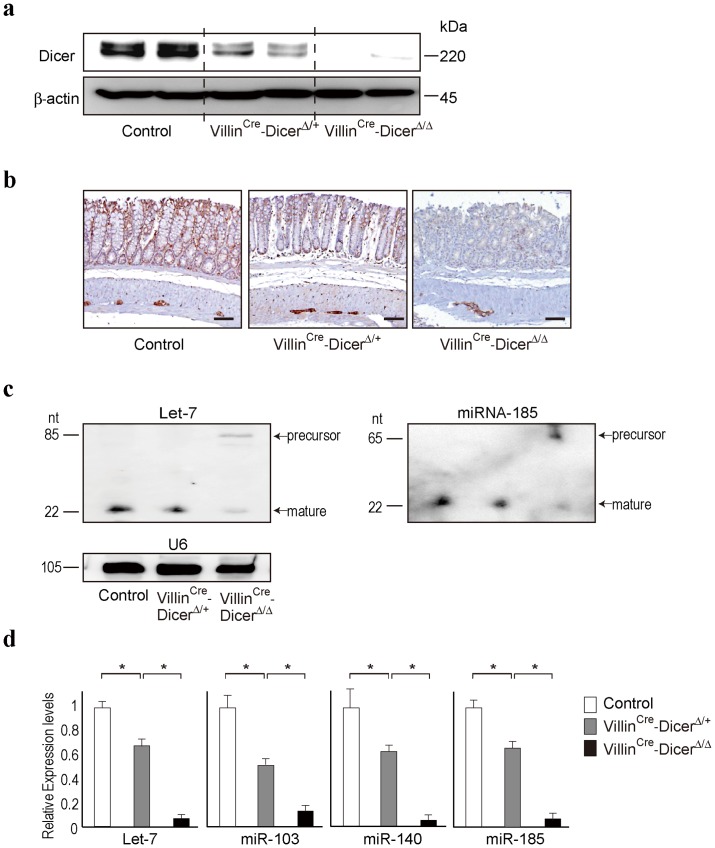
Intestinal-epithelial-cell-specific Dicer gene ablation. **a**, Dicer protein expression levels in the isolated primary colonic epithelial cells of Dicer^fl/fl^ control, Villin^Cre^-Dicer^Δ/+^, and Villin^Cre^-Dicer^Δ/Δ^ mice. Representative western blotting images of three independent experiments are shown. **b**, Representative immunohistochemical images of DICER protein expression (in brown) in the colons of control and mutant mice. The sections are 30 mm proximal to the anal canal. Scale bars  = 200 µm. Similar results were obtained from at least three independent mice per group. **c, d**, The expression levels of the indicated miRNAs in isolated colonic epithelial cells of control and mutant mice determined by Northern blotting (c) and qRT-PCR (d). Representative images of three independent experiments are shown (c). U6 was used as the loading control. The data were obtained from three independent experiments and are shown as means ± s.d. after adjusting the value of control mice as 1. *, *p*<0.05 (d).

To determine the function of *Dicer* and its downstream miRNAs in intestinal epithelia, we generated mutant mice lacking *Dicer* specifically in intestinal epithelial cells (Villin^Cre^-Dicer^Δ/Δ^) by crossing *loxP*-flanked (Dicer^fl/fl^) mice with villin promoter driven-Cre (Villin^Cre^)-expressing mice. As comparisons, we used Dicer^fl/fl^ control mice and also included Villin^Cre^-Dicer^Δ/+^ heterozygous mice. While residual DICER protein may be derived cells surrounding the intestinal epithelium that were not completely removed during cell isolations, we confirmed dramatically reduced expression levels of DICER protein in the isolated colonic epithelial cells of Villin^Cre^-Dicer^Δ/Δ^ mice (Figure 1a). In contrast, the expression levels of DICER in colonic epithelial cells of Villin^Cre^-Dicer^Δ/+^ heterozygous mice were intermediate to those of Dicer^fl/fl^ control and Villin^Cre^-Dicer^Δ/Δ^ mice (Figure 1a). Immunohistochemical analyses of DICER expression using colon tissues of control and *Dicer1*-mutant mice showed similar DICER expression levels in epithelial cells (Figure 1b). The functional levels of DICER, determined by the abundance of representative mature miRNAs in the colon epithelia of control and mutant mice, appeared to proportionally reflect the deletion of *Dicer1* alleles (Figure 1c and d). These results suggest that *Dicer* was successfully ablated in the intestinal epithelial cells of Villin^Cre^-Dicer^Δ/Δ^ homozygous and Villin^Cre^-Dicer^Δ/+^ heterozygous mice, and also suggest that the expression levels of DICER and subsequent function of miRNA maturation were proportionally dependent on the number of mutant alleles.

### Histological changes of the intestine in intestinal epithelia-specific *Dicer1*-mutant mice

**Figure 2:**
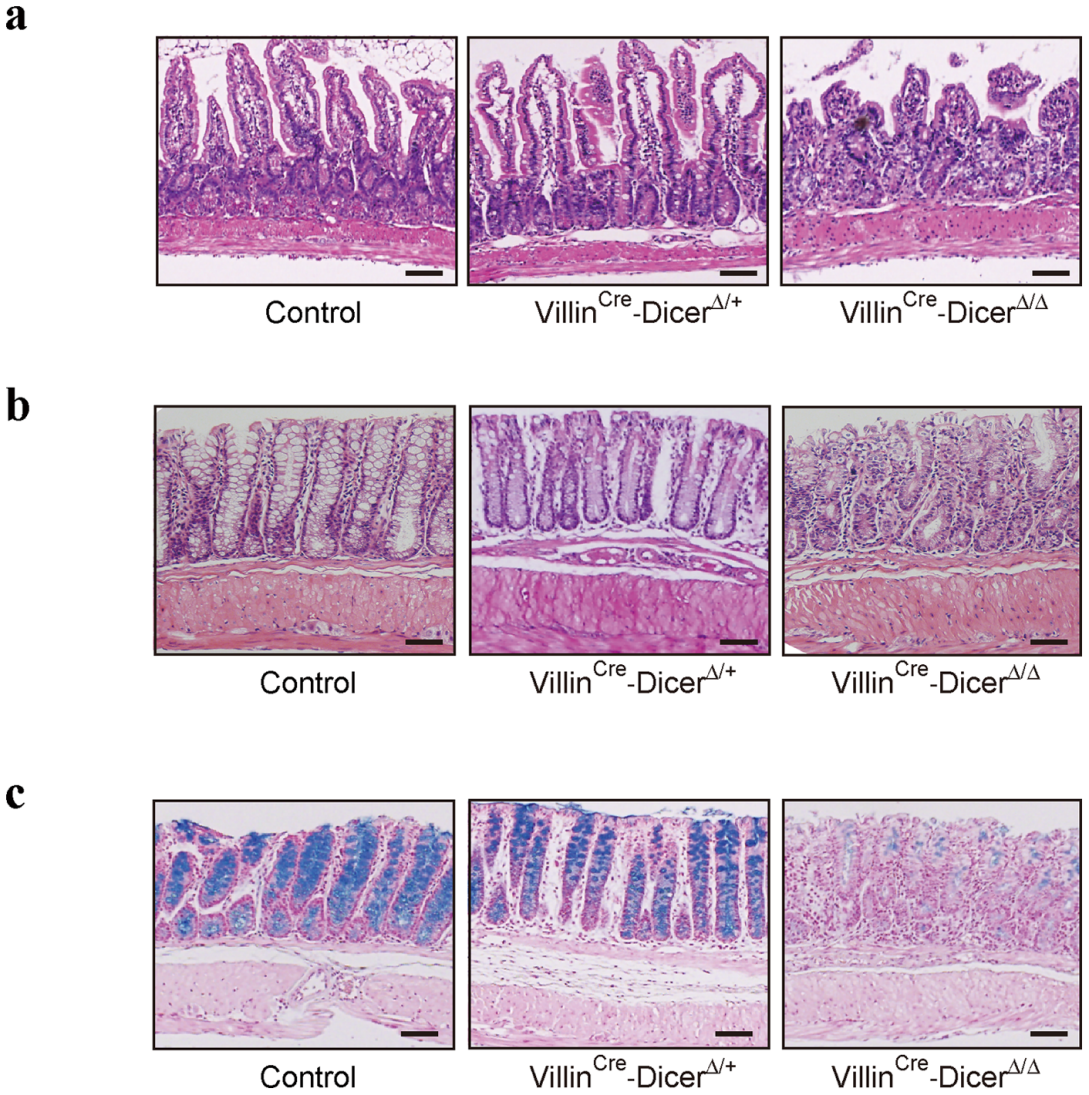
Phenotypic changes in the intestine of untreated Villin^Cre^-Dicer^Δ/Δ^ mice. **a,** Hematoxylin and eosin staining of small intestine tissues 40 mm proximal to the ileo-colonic junction. Villin^Cre^-Dicer^Δ/Δ^ mice displayes the most deregulated crypt morphologies. Scale bars  = 200 μm. Similar results were obtained from at least five mice per group. **b, c**, Hematoxylin and eosin staining (b) and alcian blue staining (c) of colonic tissues 30 mm proximal to the anal canal. Villin^Cre^-Dicer^Δ/Δ^ mice displayes the most disorganized lamina propria structure with increased inflammatory cell infiltration and the fewest goblet cells. Scale bars  = 200 μm. Similar results were obtained from at least five mice per group.

The morphological changes of the intestine in *Dicer1*-mutant mice were determined using intestinal tissues derived from 12 week old animals. Consistent with a previous report [22], disorganized morphologies of the small intestine lamina propria in *Dicer1*-mutant mice were clearly observed (Figure 2a). In the large intestine, the *Dicer1*-mutant mice had disorganized crypt structure (Figure 2b). These deregulated phenomena were most remarkable in Villin^Cre^-Dicer^Δ/Δ^ homozygous mice. We also observed increased inflammatory cell infiltration in the lamina propria in *Dicer1*-mutant mice that was most remarkable in Villin^Cre^-Dicer^Δ/Δ^ homozygous mice (Figure 2b and Figure S1). Furthermore, the degree of inflammatory cell infiltration was less in Villin^Cre^-Dicer^Δ/+^ heterozygous mice than in homozygous mice, but more than in Dicer^fl/fl^ control mice (Figure 2b). While *Dicer1*-mutant mice had fewer goblet cells (Figure 2c), consistent with previous reports [22], [23], Villin^Cre^-Dicer^Δ/+^ heterozygous mice showed an intermediate number of goblet cells in control and homozygous mice (Figure 2c). These results suggest that histological changes in intestinal morphology caused by *Dicer* mutants are dependent on the expression levels of DICER protein and subsequent expression levels of mature miRNAs.

### Comparable severity of experimental inflammation in the colon of *Dicer1*-mutant mice

**Figure 3:**
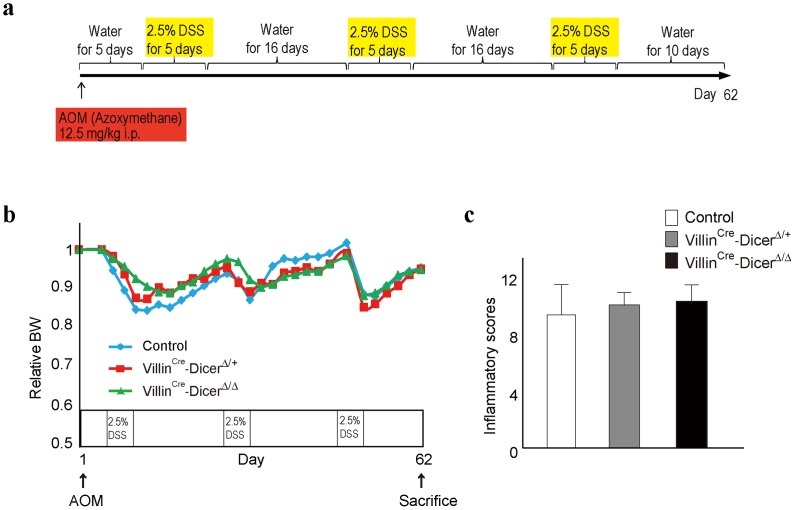
Comparable severity of inflammation in the colon of Villin^Cre^-Dicer^Δ/Δ^ mice. **a**, The protocol for experimental inflammation-associated colon tumorigenesis used in this study. **b**, Body weight changes of the Dicer^fl/fl^ control mice (n = 8), Villin^Cre^-Dicer^Δ/+^ mice (n = 8), or Villin^Cre^-Dicer^Δ/Δ^ mice (n = 8) during DSS-induced colon inflammation. Changes in body weight were measured every 2 days. **c**, Inflammatory scores of mice in each group (n = 8 per group) at day 62 are shown. Data are presented as means ± s.d.

To determine the biological effects of DICER and mature miRNAs in the intestinal epithelial cells on chronic colitis-induced tumorigenesis, we treated eight 12 week-old mice with a single dose of azoxymethane (AOM, 12.5 mg/kg) followed by three cycles of DSS administration in the drinking water (Figure 3a) as a tumor model [24]. AOM is a procarcinogen, which upon metabolic activation causes formation of O^6^-methyl-guanine [25]. Repeated DSS administration causes chronic inflammation, which greatly enhances the incidence of AOM-induced tumors [24]. During the process of repeated inflammation induction, we examined if the severity of inflammation in the intestine differed among *Dicer1*-mutant mice. The severity of induced inflammation was determined by the degree of body weight loss, the colon length at the end point, and the histological inflammation scores after one round of DSS treatment, all of which are commonly used to measure the severity of inflammation in this model [26]. Although infiltration of inflammatory cells into the epithelia were observed before induction of experimental inflammation in Villin^Cre^-Dicer^Δ/Δ^ homozygous mice, the severity of inflammation after the induction in this model was comparable in control and *Dicer1*-mutant mice (Figure 3b, c and Figure S2). These results suggested that the inflammation-inducing model used here was severe enough to result in similar inflammation severities in the intestine irrespective of the mutant types.

### *Dicer* heterozygous mice were most liable to colitis-associated tumors

**Figure 4:**
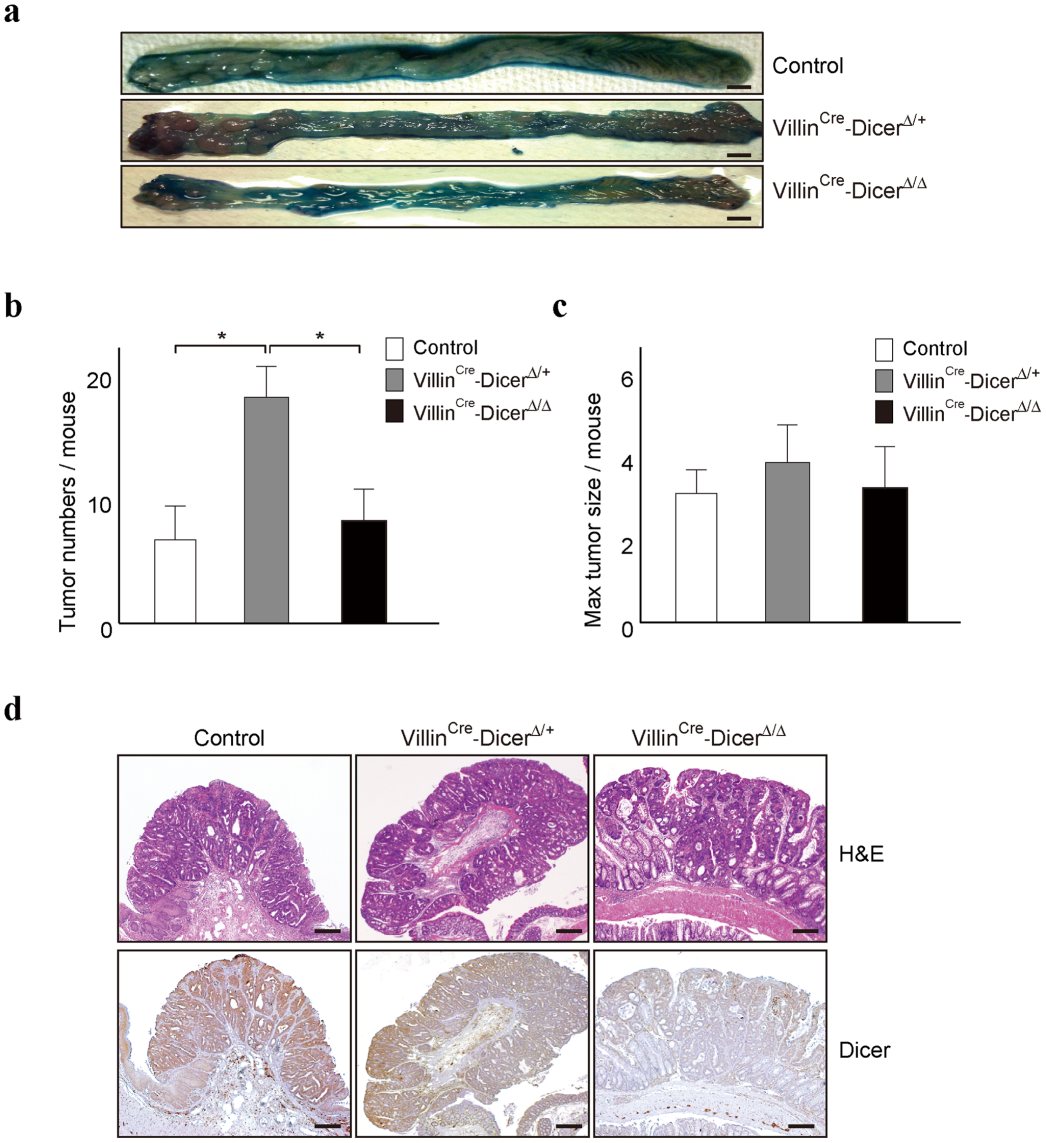
Heterozygous mice were more liable to colitis-associated tumors. **a, b**, Increased number of tumors in Villin^Cre^-Dicer^Δ/+^ mice. Representative gross colon images are shown in (a). The mean number of tumors per mouse was calculated. Data are presented as means ± s.d. (n = 8 per group) in (b). *, *p*<0.05. **c**, The maximum tumor size per mouse was measured. Data are presented as means ± s.d. (n = 8 per group). **d**, Hematoxylin and eosin staining of colon tumors (upper panels) and immunohistochemical analyses of DICER expression in similar sections of tumors (lower panels) from control and *Dicer1*-mutant mice after induction of colitis-associated tumors. Scale bars  = 200 μm. Representative images are shown from three independent mice per group.

While global down-regulation of miRNAs in many tumors have been reported [1], [4], [27], it was also reported that heterozygosity for the *Dicer1* allele, but not complete loss of *Dicer1* alleles, enhances tumor development in K-*ras*-driven lung cancer and *RB*-driven retinoblastoma models [18], [19]. To determine whether this unique haploinsufficiency concept is applicable to inflammation-associated tumors in the intestine, we compared the number of colon tumors in the AOM-DSS colitis-associated tumor models in three mouse groups (Dicer^fl/fl^ control mice, Villin^Cre^-Dicer^Δ/+^ heterozygous mice, and Villin^Cre^-Dicer^Δ/Δ^ homozygous mice). The number of colon tumors at day 62 was significantly greater in Villin^Cre^-Dicer^Δ/+^ heterozygous mice. That is, while seven or eight tumors per mouse in average were observed in Dicer^fl/fl^ control mice or Villin^Cre^-Dicer^Δ/Δ^ homozygous mice, more than fifteen tumors per mouse were observed in Villin^Cre^-Dicer^Δ/+^ heterozygous mice (Figure 4a and b). Because inflammation severities were almost similar irrespective of mutant type, the differences in tumor numbers observed here were likely not due to differences in the degree of inflammation. In addition, maximum tumor size was not significantly different (Figure 4c) and the histological tumor appearances were also not clearly different in these three groups (Figure 4d). These results suggest that the unique haploinsufficiency of *Dicer* is also applicable to inflammation-associated colon tumorigenesis.

To examine the possibility of non-autonomous effects of DICER on inflammation associated-tumorigenesis and to confirm the expression levels of DICER in the resultant tumors, the expression levels of DICER protein in tumors were determined by immunohistochemistry. The expression levels of DICER protein in tumors were approximately proportional to the number of remaining wild-type *Dicer1* alleles and similar to those in the non-tumor colonic epithelial cells (Figure 4d and Figure S3). These results suggest that the effects of DICER expression levels on inflammation-associated tumorigenesis are likely cell-autonomous.

### Expression levels of tumor-related genes are associated with DICER expression levels

**Figure 5:**
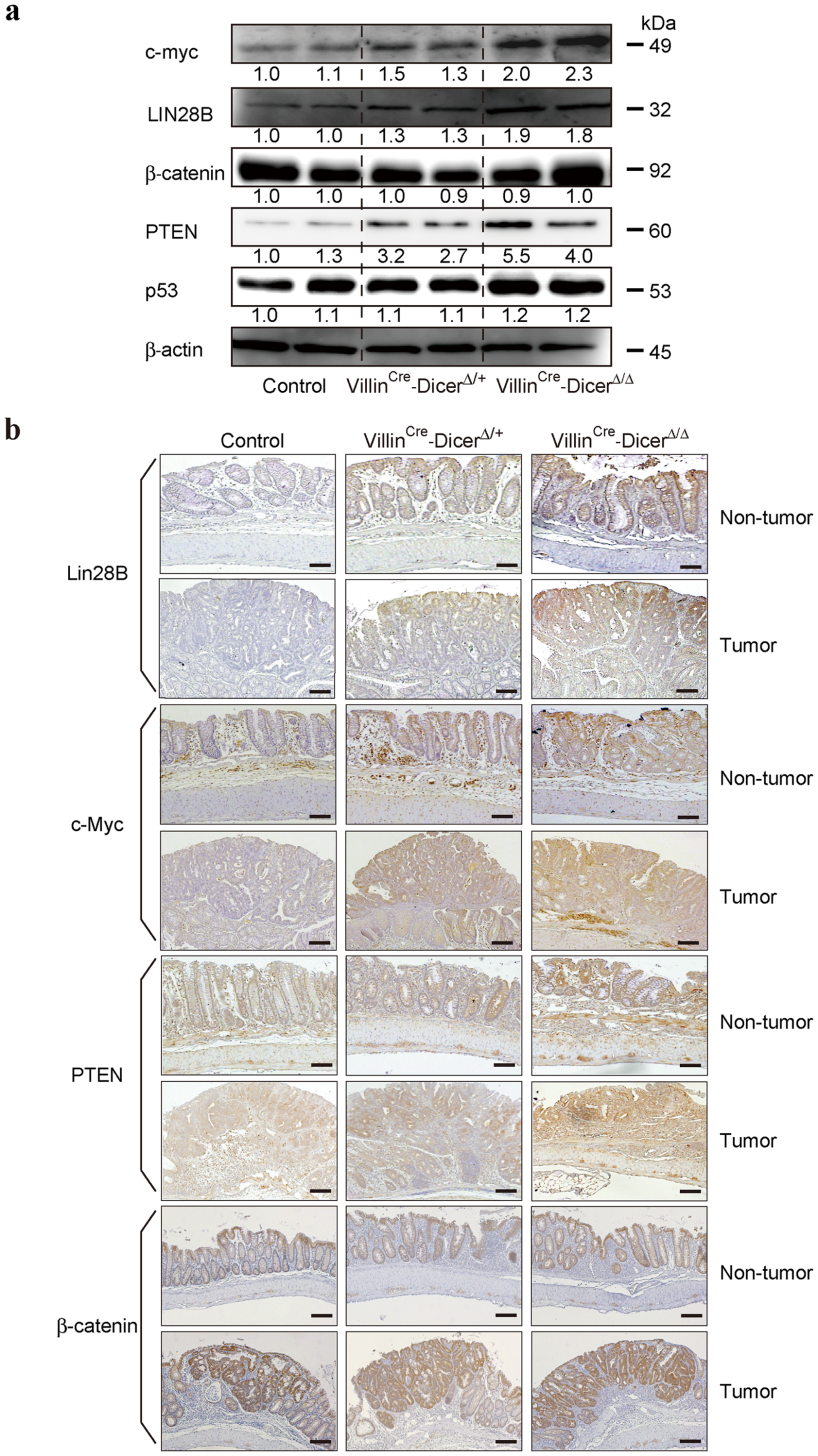
Expression levels of representative genes in colitis-associated tumors in *Dicer1*-mutant mice. **a**, Western blotting for the indicated genes in isolated colon epithelial cells in bulk after induction of colitis-associated tumors. Representative images of two independent mouse sets are shown. The band intensities were quantitated and adjusted by the expression levels of β-actin. The calculated ratios are indicated below each panel after setting the value of the control mouse as 1.0. **b**, Immunohistochemical analyses of the indicated protein expression in the tumor and surrounding non-tumor tissue from control and *Dicer1*-mutant mice after induction of colitis-associated tumors. Scale bars  = 200 μm. Representative images are shown. Similar results were obtained from four independent mice per group.

To gain insights into the genes responsible for this *Dicer* haploinsufficiency in colitis-associated tumorigenesis, we determined the expression levels of representative genes known as oncogenes or tumor suppressors in colon tumorigenesis. Similar to the patterns of DICER protein expression levels in *Dicer1* mutants, the expression levels of LIN28B and c-myc, known as oncogenes in colon tumors [28]–[30] regulated by miRNAs [29], [31] were inversely related with the number of wild-type *Dicer* alleles in both tumors and non-tumor parts of the colonic epithelia (Figure 5a and b and Figure S3). The expression levels of PTEN, a known tumor suppressor gene in the colon, were also dependent on the number of intact *Dicer1* alleles in both tumors and non-tumor tissues (Figure 5a and b and Figure S3). However, the expression levels of β-catenin and p53 were not dramatically changed in the *Dicer* mutants used in this study (Figure 5a and b and Figure S3). These results suggest that the expression levels of most oncogenes and tumor suppressor genes are proportional to DICER expression levels in both tumors and non-tumor tissues, but the magnitude of expression level change is gene-specific.

## Discussion

In this study, we report the effects of *Dicer* deletion on intestinal epithelia using intestinal-epithelial-cell-specific *Dicer1* ablated mice. In untreated mice, DICER in the intestinal epithelial cells is crucial for the maintenance of mucosal morphology in the small intestine and the differentiation of goblet cells in the large intestine, consistent with a previous report [22], [23]. These observed phenotypes were most severe in homozygous Villin^Cre^-Dicer^Δ/Δ^ mice. However, heterozygous Villin^Cre^-Dicer^Δ/+^ mice were most prone to tumors, while *Dicer1*-null mice developed marginal tumorigenesis, suggesting that the promotion of tumorigenesis is conditional based on the number of wild-type *Dicer1* alleles.

Global down-regulation of miRNAs is frequently observed in cancers [1], [4], [5] and deregulation of the miRNA processing pathway is also associated with many types of cancers, which may, in turn, cause aberrations in global miRNA profiles [6]–[11]. MiRNA-processing genes, such as DICER, TRBP and XPO5, are frequently deleted in humans [6]. However, no homozygous deletion of such genes in tumors has been reported and complete loss of *Dicer* did not preclude tumor formation in *in vivo* experimental models [21]. Therefore, partial loss of mature miRNAs may be advantageous to tumorigenesis while complete loss may be a disadvantage. From this point of view, partial loss of the expression or functions of miRNA processing pathway molecules, including DICER, may be crucial during tumorigenesis.

The precise mechanisms underlying how monoallelic loss of *Dicer* leads to greater colitis-associated tumorigenesis are still unknown. Because the severity of inflammation was not significantly different in the model used here, the differences in inflammation severity as a cause of tumorigenesis were not considerable. Cells that have escaped *Dicer* deletion were the primary source of induced hepatocarcinoma in a liver cancer model [32], which is consistent with less tumorigenesis with a complete loss of *Dicer*. We hypothesized that *Dicer* loss may autonomously affect the surrounding cells with residual DICER expression. However, in the colitis-associated tumors examined here, the expression levels of DICER in the tumors as well as in the non-tumor tissues were as expected from the allelic numbers of the disrupted *Dicer1.* Thus, the effects of *Dicer1* disruption were likely cell-autonomous.

The expression levels of representative known tumor suppressors and oncogenes determined in this study were primarily inversely proportional to the DICER expression levels. However, because miRNA regulation of gene expression varies from gene to gene, the impaired delicate balance between oncogenes and tumor suppressive genes may be involved in the unique haploinsufficiency of *Dicer* in tumorigenesis. While further study is necessary to determine the molecular background of *Dicer* haploinsufficiency, we consider that the following possibilities should also be elucidated: 1) Genes other than those investigated here are involved in tumorigenesis in this model; 2) DICER may have effects on tumorigenesis through unknown functions other than gene expression regulation through miRNA processing; 3) the accumulated precursors or repetitive RNAs, such as Alu, which could not be processed due to insufficient DICER expression may have effects on the tumorigenesis. These questions should be determined in the future to clarify the unique role of the deregulation of DICER and related deregulation of miRNAs in tumorigenesis.

Untreated *Dicer1*-mutant mice showed decreased goblet cells and increased inflammatory cell infiltration in the large intestine. DICER and its subsequent miRNA pathway may be related to the differentiation of intestinal epithelial cells, particularly goblet cells [22], [23] and through the deregulation of the epithelial cell differentiation, changes in the microbiome of the gut may lead to infiltration of inflammatory cells into the intestinal mucosa [23]. It cannot be denied that these chronic stress conditions in Villin^Cre^-Dicer^Δ/Δ^ mice may also be, to some extent, related to the observed results of *Dicer* haploinsufficiency in the colitis-associated tumorigenesis.

In conclusion, using intestinal-epithelial-cell-specific *Dicer* ablated mice, we identified the unique haploinsufficient role of *Dicer* in colitis-associated tumorigenesis. It is generally accepted that complete loss of tumor suppressor function is necessary for tumor development. However, in this study, monoallelic loss of *Dicer* in intestinal epithelial cells accelerates tumor formation in colitis-associated tumorigenesis, but complete loss of *Dicer* did not, which is consistent with previous reports of the role of *Dicer* in other organs [18]–[20]. These results suggest that complete *Dicer* inactivation is deleterious for cancer development, while its partial inactivation promotes tumor formation. Although it is clear that the effects of aberrations in the miRNA pathway play a crucial role in tumorigenesis, this unique haploinsufficient role of *Dicer* needs further analysis to elucidate the intrinsic complex mechanisms and to apply this phenomenon to novel translational applications.

## Methods

### Mice

Eight-week-old C57BL/6 mice were purchased from CLEA Japan (Tokyo, Japan). Villin^Cre^ and Dicer^f/f^ mice were purchased from the Jackson Laboratory (Bar Harbor, ME). Ablation of DICER protein expression was confirmed by antibodies raised against the C-terminal portion of the DICER protein (sc-30226, Santa Cruz Biotechnology, Santa Cruz, CA). All experimental protocols were approved by the Ethics Committee for Animal Experimentation at the Graduate School of Medicine, the University of Tokyo, Japan and conducted in accordance with the Guidelines for the Care and Use of Laboratory Animals of the Department of Medicine, the University of Tokyo.

### Inflammation associated-colon tumor model

Mice (8–12 weeks old) were injected intraperitoneally with 12.5 mg/kg azoxymethane (AOM; Wako, Osaka, Japan). After 5 days, 2.5% dextran sulfate sodium (DSS; ICN Biomedicals, Irvine, CA) was mixed in the drinking water for 5 days, followed by 16 days of regular water. This cycle was repeated twice and mice were sacrificed 10 days after the last cycle. Induced colon tumors were counted and sized. Body weight was checked every 2 days. Histological inflammatory scoring of the colon tissues was performed in a blinded manner as reported previously [26]. The macroscopic images were taken after Indigo carmine staining.

### Isolation of primary intestinal epithelial cells

Intestinal epithelial cells were isolated using a slightly modified rapid low-temperature method [33]. Briefly, the entire colon was removed and washed with ice-cold PBS. The intestine was divided into 2–3 mm-long fragments and transferred into chelating buffer (27 mM trisodium citrate, 5 mM Na_2_PO_4_, 96 mM NaCl, 8 mM KH_2_PO_4_, 1.5 mM KCl, 0.5 mM DTT, 55 mM D-sorbitol, 44 mM sucrose, 6 mM EDTA, 5 mM EGTA, pH 7.3) for 45 min at 4°C. Intestinal epithelial cells were dissociated by repeated vigorous shaking. Tissue debris was removed using a 100 µm cell-strainer and cells were collected by centrifugation at 150×*g* for 10 min at 4°C. The viability of cells was confirmed by trypan blue staining and cells processed for protein extraction.

### Quantitative RT-PCR and Northern blotting for miRNAs

Quantitative RT-PCR analysis of miRNA expression was performed as described previously [34]. Northern blotting analysis of miRNAs was performed as described previously [35]. Briefly, total RNA was extracted using TRIzol Reagent (Invitrogen, Carlsbad, CA) according to the manufacturer's instructions. Five μg of small RNA were resolved in denaturing 15% polyacrylamide gels containing 7 M urea in 1× TBE and transferred to Hybond N+ membrane (GE Healthcare, Waukesha, WI) in 0.25× TBE. Membranes were UV-crosslinked and prehybridized in hybridization buffer. Hybridization was performed overnight at 42°C in ULTRAhyb-Oligo Buffer (Ambion, Austin, TX) containing a biotinylated probe specific for let-7b and miR-185, which had previously been heated to 95°C for 2 min. Membranes were washed at 42°C in 2× SSC containing 0.1% SDS, and the bound probe was visualized using a BrightStar BioDetect Kit (Ambion). Blots were stripped by boiling in a solution containing 0.1% SDS, 5 mM EDTA for 10 min prior to rehybridization with a U6 probe with the sequence 5′-CACGAATTTGCGTGTCATCCTT′-3.

### Western blotting

Western blotting was performed as described previously [36]. The following antibodies were used: anti-DICER (SAB4200087) and anti-β-actin (A5316) were purchased from Sigma-Aldrich (St. Louis, MO). Anti-c-Myc (sc-40) and anti-DICER (sc-30226) were purchased from Santa Cruz Biotechnology. Anti-LIN28B (5422), anti-PTEN (9559), and anti-β-catenin (9582) were purchased from Cell Signaling Technology (Danvers, MA). Anti-p53 (OP03) was purchased from Calbiochem (San Diego, CA).

### Immunohistochemistry

Immunohistochemistry was performed as described previously [36]. The following antibodies were used: anti-LIN28B (16178-1-A, Proteintech Group, Chicago, IL), anti-DICER, anti-c-myc, anti-PTEN, and anti-β-catenin described above.

### Statistical analysis

Significant differences between groups were determined using Student's *t*-test when variances were equal. When variances were unequal, Welch's *t*-test was used. *P*-values <0.05 were considered to indicate statistical significance.

## Supporting Information

Figure S1 **Inflammatory scores in the colon of untreated Dicer1-mutant mice.** Inflammatory scores in the colon of untreated mice from each group (n = 4 per group). Data are presented as means ± s.d. *, p<0.05.
(PDF)

Figure S2 **Severity of inflammation after induction of colitis in** ***Dicer1*** **-mutant mice.** **a**, Representative colonic tissue images in hematoxylin and eosin staining at day 12. Control and Dicer1-mutant mice showed similar inflammation severity. The sections are 30 mm proximal to the anal canal. Scale bars  = 200 μm. Similar results were obtained from five independent mice per group. **b**, The length of the colon in mice at day 62 is shown. Data are presented as means ± s.d. (n = 8 per group).
(PDF)

Figure S3 **Immunohistochemical analyses of protein expression in control and** ***Dicer1*** **-mutant mice after induction of colitis-associated tumors.** Immunohistochemical analyses of the indicated protein expression in tumors and surrounding non-tumor tissue from control and Dicer1-mutant mice after induction of colitis-associated tumors. Scale bars  = 200 μm. Dashed lines indicate the border between the tumor and its surrounding tissues. Representative images are shown. Similar results were obtained from four independent mice per group.
(PDF)
