# Supplementary figures and images for: Unique Haploinsufficient Role of the MicroRNA-Processing Molecule Dicer1 in a Murine Colitis-Associated Tumorigenesis Model
Source: PLoS One. 2013 Sep 2;8(9):e71969. doi: 10.1371/journal.pone.0071969 (PMC3759383; doi:10.1371/journal.pone.0071969)

Supplementary Figure 1

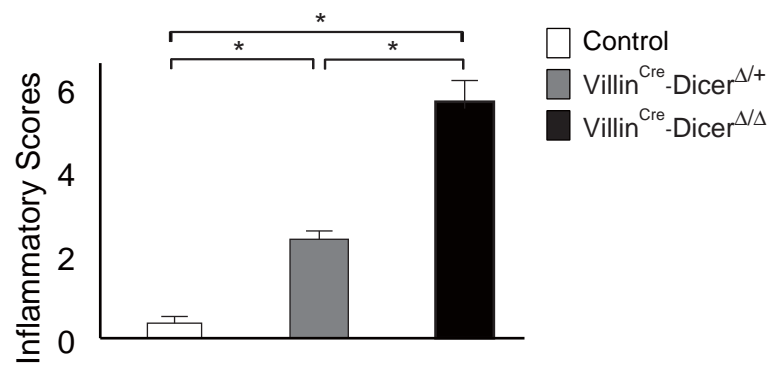

Supplement: Figure S1 — Inflammatory scores in the colon of untreated Dicer1-mutant mice. Inflammatory scores in the colon of untreated mice from each group (n = 4 per group). Data are presented as means ± s.d. *, p<0.05. (PDF) [file pone.0071969.s001.pdf]

Supplementary Figure 2

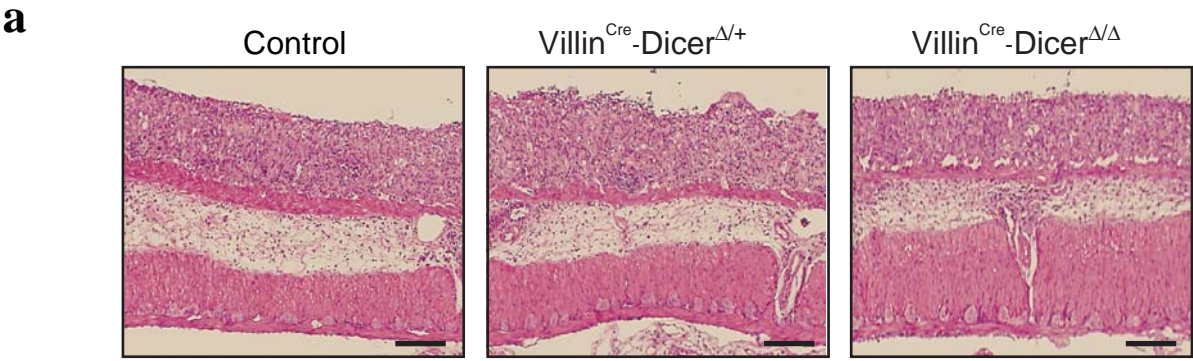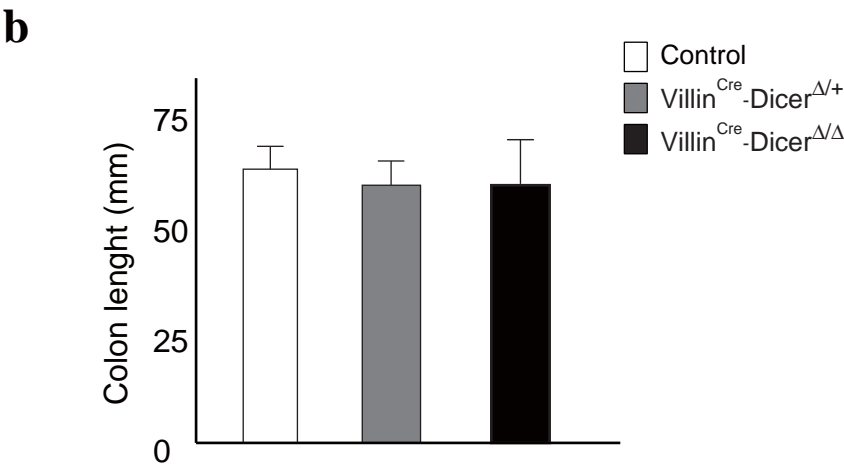

Supplement: Figure S2 — Severity of inflammation after induction of colitis in Dicer1 -mutant mice. a, Representative colonic tissue images in hematoxylin and eosin staining at day 12. Control and Dicer1-mutant mice showed similar inflammation severity. The sections are 30 mm proximal to the anal canal. Scale bars = 200 μm. Similar results were obtained from five independent mice per group. b, The length of the colon in mice at day 62 is shown. Data are presented as means ± s.d. (n = 8 per group). (PDF) [file pone.0071969.s002.pdf]

Supplementary Figure 3

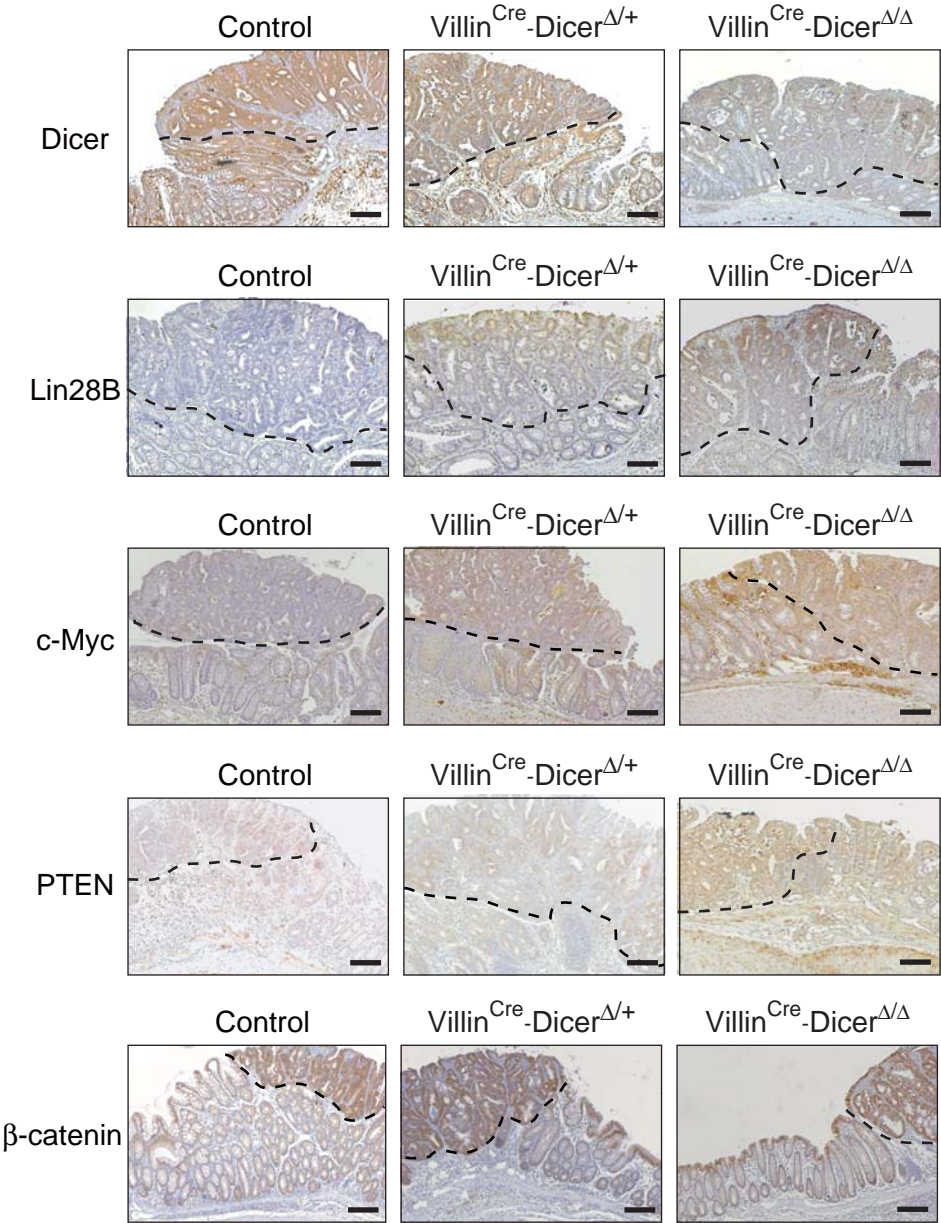

Supplement: Figure S3 — Immunohistochemical analyses of protein expression in control and Dicer1 -mutant mice after induction of colitis-associated tumors. Immunohistochemical analyses of the indicated protein expression in tumors and surrounding non-tumor tissue from control and Dicer1-mutant mice after induction of colitis-associated tumors. Scale bars = 200 μm. Dashed lines indicate the border between the tumor and its surrounding tissues. Representative images are shown. Similar results were obtained from four independent mice per group. (PDF) [file pone.0071969.s003.pdf]
